# Case report: Transcatheter tricuspid valve intervention using K-Clip™ system after prior Kay’s annuloplasty

**DOI:** 10.3389/fcvm.2023.1169524

**Published:** 2023-08-09

**Authors:** Shengjun Wu, Xiaoyi Dai, Lingshan Liu, Shuai Yuan, Peng Teng, Yiming Ni

**Affiliations:** ^1^ Department of Cardiovascular Surgery, The First Affiliated Hospital, School of Medicine, Zhejiang University, Hangzhou, China; ^2^ Department of Echocardiography and Vascular Ultrasound Center, The First Affiliated Hospital, School of Medicine, Zhejiang University, Hangzhou, China

**Keywords:** tricuspid regurgitation, K-Clip™, annuloplasty, transcatheter, tricuspid valve

## Abstract

The K-Clip™ system is emerging as an alternative to correct tricuspid regurgitation (TR) for patients with high surgical risk. However, patients with recurrent severe tricuspid regurgitation after prior Kay’s annuloplasty are not generally deemed to be candidates for K-Clip™ implantation. Herein, we report a case of a 63-year-old woman with recurrent symptomatic torrential tricuspid regurgitation 5 years after double valve replacement with Kay’s annuloplasty of the tricuspid valve. The K-Clip™ was successfully implanted, and the severity of tricuspid regurgitation and dimensions of tricuspid annulus achieved significant reduction. In conclusion, K-Clip™ can still be feasible and effective for patients with prior Kay’s annuloplasty. However, indications become more rigorous, and evaluation should be more comprehensive.

## Introduction

**Figure 1:**
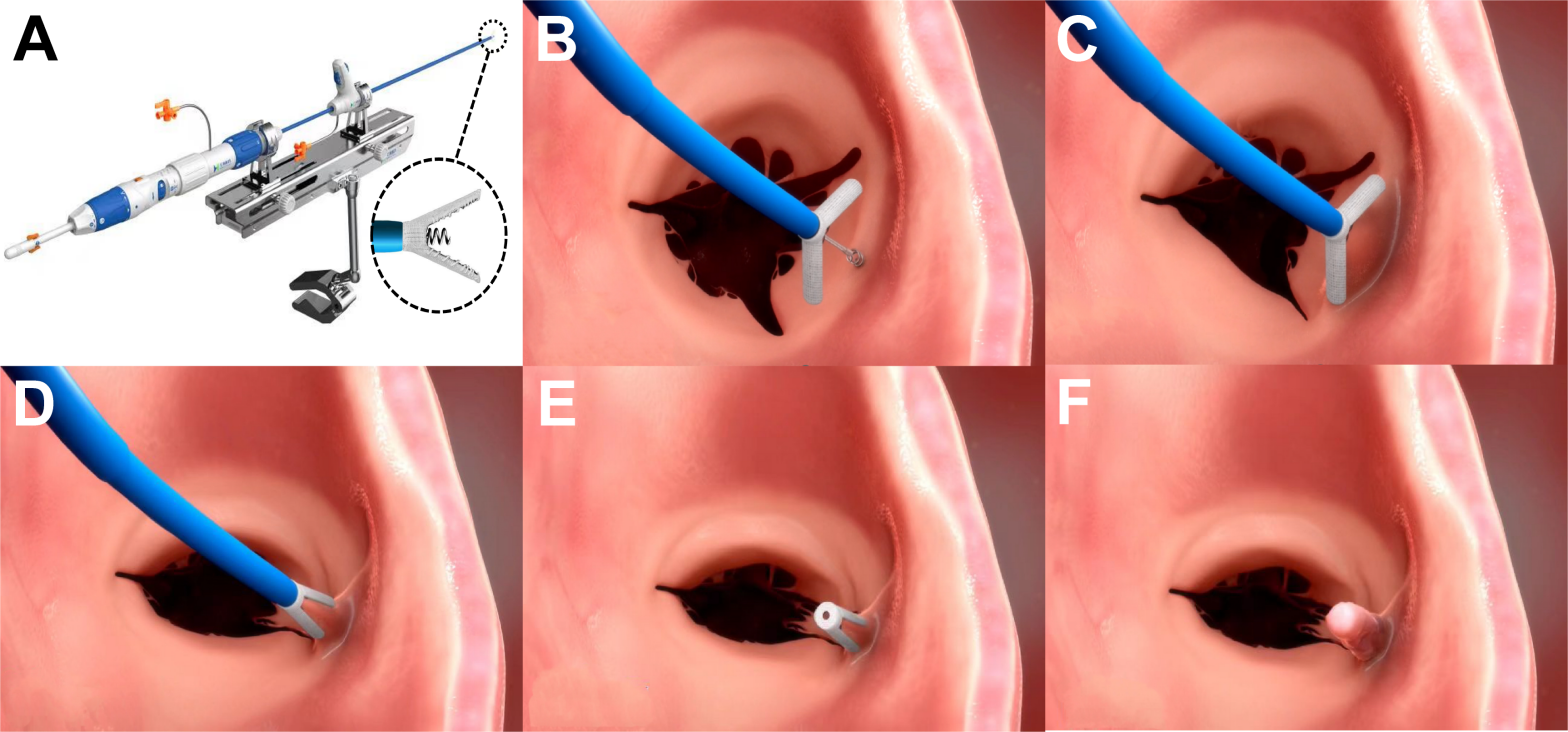
The K-Clip™ system and the main steps of implantation. (**A**) Showing the delivery system of K-Clip™, and the clip consists of clamp arms and the tapping screw-shaped anchor. (**B**) Open the clamp arms and insert the tapping screw-shaped anchor to the middle of posterior tricuspid annulus. (**C**) Pull back the annular tissue into the clip. (**D**) Clamp the clip. (**E**) Release the clip and withdraw the guidewire. (**F**) Showing endothelialization of the implanted clip. Images provided by Huihe Medical Technology with permission.

Tricuspid regurgitation (TR) is divided into two stages, primary TR and functional TR, the latter accounting for more than 90%. Most commonly, functional TR results from tricuspid annulus (TA) dilation due to left-sided heart disease and pulmonary vascular disease (1). A current guideline states that surgery is recommended in symptomatic patients with severe functional TR in the absence of severe ventricular dysfunction and pulmonary hypertension (2). However, isolated tricuspid valve surgery has been seldom performed due to its reported high mortality rates (3). In recent years, various transcatheter tricuspid valve intervention (TTVI) devices are emerging as an alternative for patients with prohibitive surgical risk (4), among which the K-Clip™ system (Figure 1) is designed to mimic the Kay’s annuloplasty and achieve posterior annular reduction and bicuspidization of the tricuspid valve (5).

Generally, patients with recurrent severe functional TR after prior Kay’s annuloplasty are not deemed to be candidates for K-Clip™ implantation. Nevertheless, based on the experience of more than 40 cases of K-Clip™ implantation in our institution, we believe that it can also achieve acceptable outcomes in this specific population. Herein, we introduced a typical case and the process of diagnosis and treatment.

## Case presentation

The patient was a 63-year-old woman with a history of rheumatic heart disease, involving moderate mitral stenosis with regurgitation, moderate aortic stenosis with regurgitation, and mild TR. Five years ago, she had undergone double valve replacement combined with Kay’s annuloplasty of the tricuspid valve.

The patient complained of worsening chest tightness and peripheral edema in recent half a year. Upon admission, the physical examination revealed significant edema of both lower extremities, arrhythmia, and moderate murmur in the auscultation area of the tricuspid valve. The electrocardiogram showed atrial fibrillation. The transthoracic echocardiography suggested a normal function of double mechanical prostheses and torrential functional TR. Anticoagulation was achieved by an appropriate dose of warfarin, and the international normalized ratio was 2.32. The patient was evaluated as being at high risk for redo surgical tricuspid annuloplasty and was considered for TTVI using the K-Clip™ system (Huihe Medical Technology, Shanghai, China).

**Figure 2:**
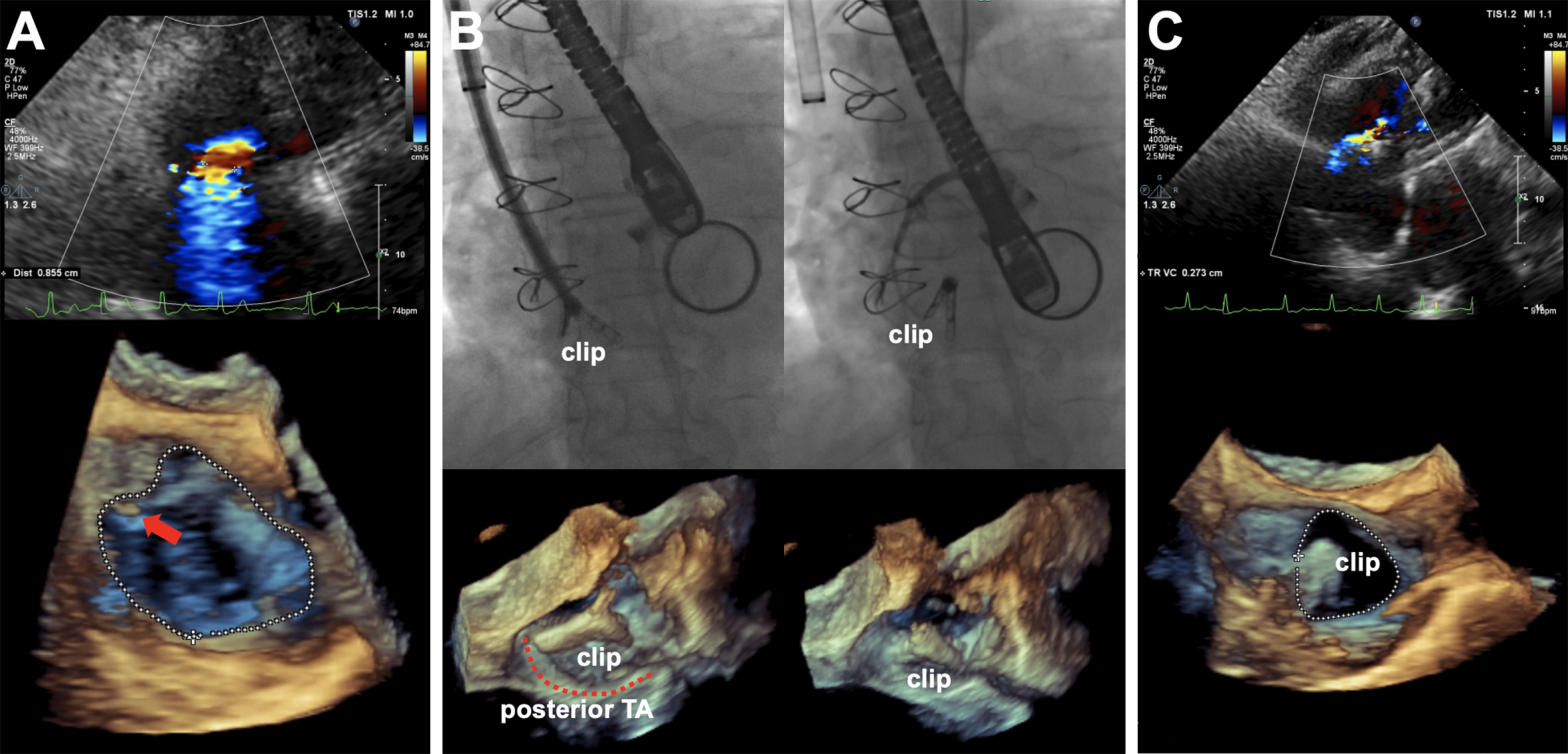
(**A**) Intraprocedural TEE showing torrential TR with EROA of 0.74 cm^2^, regurgitation volume of 51 ml, and vena contracta width of 0.71 cm (upper panel) and 3D work plane revealing the original knot of the Kay’s procedure (red arrow) and TA morphology before K-Clip™ implantation (lower panel). (**B**) Real-time fluoroscopy (upper panel) and 3D work plane of TEE (lower panel) showing the opening clamp and the clip on the target location after release. (**C**) Postprocedural TEE showing an immediate reduction of TR grade with an EROA of 0.16 cm^2^, regurgitation volume of 12 ml, and vena contracta width of 0.27 cm (upper panel) and 3D work plane (lower panel) showing the TA morphology after K-Clip™ implantation (red arrow). TEE, transesophageal echocardiogram; TR, tricuspid regurgitation; EROA, effective regurgitant orifice area; TA, tricuspid annulus.

**Figure 3:**
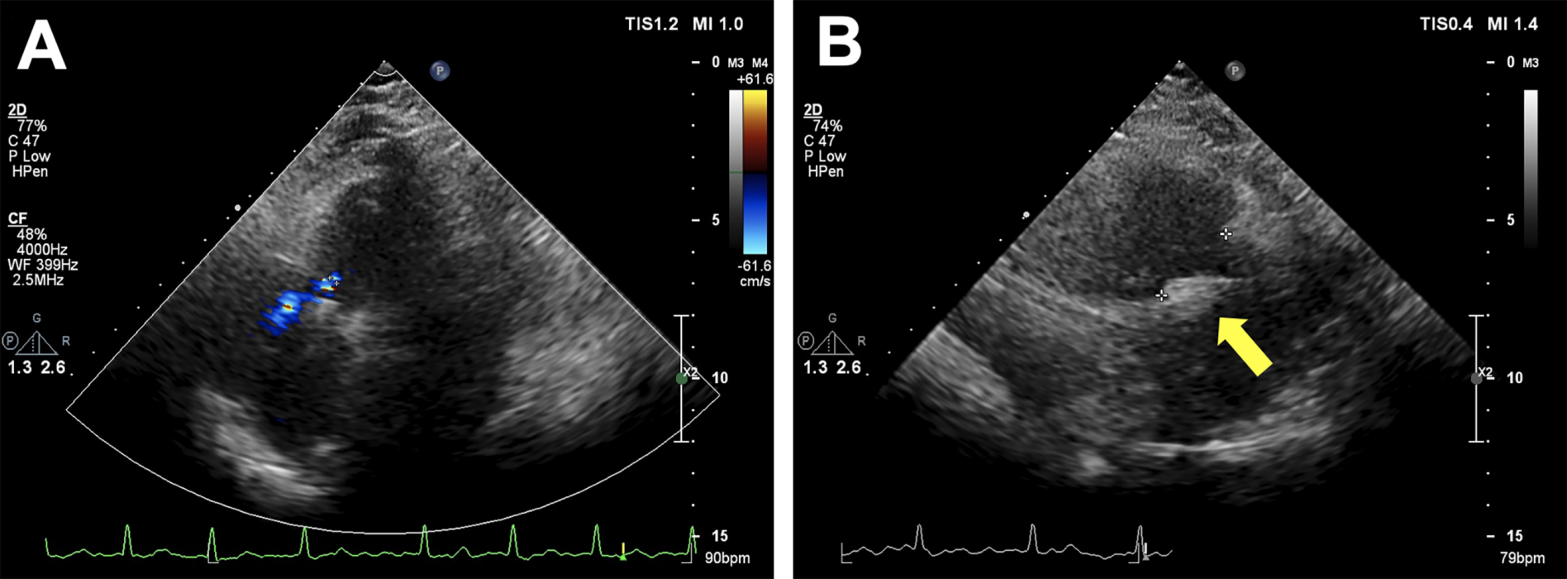
Transthoracic echocardiography after 3 months of follow-up. (**A**) Showing mild residual tricuspid regurgitation. (**B**) Showing the secure position of the device (yellow arrow).

The patient was horizontally positioned and under general anesthesia. A 16-mm-clip arm would be preprocudurally applied according to the comprehensive evaluation of the patient’s TA. The K-Clip™ procedure was performed with the real-time monitor of fluoroscopy and transesophageal echocardiography (TEE). The TEE confirmed the torrential TR (vena contracta width of 0.71 cm), as well as that the distance between the target sites for clip implantation and the knot of the prior Kay’s procedure (Figure 2A) was approximately 18–20 mm, which was enough space for a 16 mm clip to clamp the posterior annular tissue. The K-Clip™ system was percutaneously inserted via the right jugular vein and steered toward the posterior TA. Then, the tapping screw-shaped anchor was inserted through the target location, and the clip was tangentially opened and oriented to the annulus, slowly pulled back with the surrounding TA tissue, and then clamped the clip, achieving tissue plication and re-bicuspidization (Figure 2B). A significant reduction in the TR grade (torrential to mild, vena contracta width of 0.25 cm) and TA dimensions was immediately observed (Figure 2C). Throughout the whole procedure, it was carefully confirmed that the original knot was not loose and meanwhile the right coronary artery flow was not interfered by the manipulation. Subsequently, the patient had an uneventful recovery and was discharged 2 days after the procedure. After 3 months of follow-up, repeated transthoracic echocardiography demonstrated mild TR (Figure 3A) and the secure position of the device (Figure 3B). Additionally, the patient reported that the previous symptoms were eliminated.

## Discussion

Isolated tricuspid valve surgery should be considered in symptomatic patients with severe functional TR (with or without prior left-sided valve surgery) who have right ventricular dilation and preserved ventricular function (2). Nowadays, surgical tricuspid valve repair using an annuloplasty ring to reduce the TA dimension under cardiopulmonary bypass remains the mainstream, but it carries high risks for elderly patients and those who require reoperation or have ventricular dysfunction. As for these patients, TTVI is a feasible option for treating functional TR with acceptable safety and simplicity (4).

Indirect annuloplasty devices of TTVI, such as TriClip and PASCAL system, aimed to restore leaflet coaptation by edge-to-edge tricuspid valve repair through a transcatheter approach, have been reported effective and safe to address functional TR (6, 7). As for direct annuloplasty of TTVI devices, Cardioband has been the only system to be approved of clinical use yet. The Cardioband system is delivered through a transfemoral approach, and the Dacron band is fixed on the TA using a series of anchors deployed from the anteroseptal to posteroseptal commissure, which was contracted afterward by a size-adjustment tool to achieve annular dimension reduction (8). Two-year outcomes of TRI-REPAIR study (NCT02981953) about Cardioband showed its favorable results in patients with symptomatic, moderate functional TR (9). Similarly, the K-Clip™ system is a transcatheter-direct annuloplasty device mimicking the Kay’s annuloplasty, which plicates the posterior annular tissue via a clip to achieve TA reduction and bicuspidization of the tricuspid valve. The first-in-human study of K-Clip™ has demonstrated acceptable procedural safety and efficacy (5, 10). Of note, it is critical to utilize the TEE and fluoroscopy to verify the target site and angle for the clip implantation. If the tapping screw-shaped anchor is inserted too close to the atrial side, the anchor might cause atrial wall perforation and further lead to less or no reduction in annular size, and it might cause leaflet tear when pulled back if the anchor is inserted too close to the leaflet. What’s more, the right coronary artery flow might be interfered by the clamping clip, so a coronary angiogram should be immediately performed after the implantation.

It is generally recognized that K-Clip™ may not be suitable for patients with recurrent TR after prior Kay’s annuloplasty, the reasons are as follows: (1) doubtful space for K-Clip™ implantation due to posterior annular plication and bicuspidization of the tricuspid valve by previous Kay’s procedure and (2) the scars caused by prior Kay’s procedure potentially interfering K-Clip™ implantation. Nonetheless, in our clinical practice, we have observed that in this population the prior Kay’s procedure often appears abnormal, presenting annular re-dilation or failure of the tricuspid valve bicuspidization due to a loose knot. In some patients, although the bicuspidization of the tricuspid valve remained intact, massive TR can be observed between the anteroposterior commissure due to significant dilation in local annular area. For this kind of patients, using the K-Clip™, TTVI may still be an effective treatment option.

The feasibility of K-Clip™ implantation for this population can be determined by evaluating whether there is an overlap with the original knot of the prior Kay’s procedure when the clip clamps. Combined with the TEE and fluoroscopy, the K-Clip™ system can simulate the clamping process and analyze where to implant the clip to achieve an effective reduction in both vena contracta width and TA size, as well as provide a guide for choosing the appropriate size of one or more clips. In this case, the preprocedural evaluation showed that the distance between the target site and the original knot was approximately 18–20 mm, which was enough for a 16-mm-size clip implantation to avoid overlapping with the original knot. After the clip was clamped, the TR grade was immediately reduced from “torrential” to “mild.” Luckily, we encountered the optimal circumstances that one clip perfectly achieved annular and TR reduction with no overlap with the original knot. Moreover, if a smaller-sized clip can avoid overlapping with the original knot but expected effect cannot be achieved, the second or more clips to be implanted in the dilated annulus should be considered to achieve optimum results. However, the K-Clip™ would be prohibited if all sizes of the clip overlapped with the original knot. In addition, a posterior TA length that exceeds 36 mm may not achieve meaningful TR reduction (more than one grade) because the longest clip arm available of the K-Clip™ is 18 mm, and, correspondingly, the maximal annular reduction length of K-Clip™ is only 36 mm. Moreover, the K-Clip™ may not be a valid choice for TR that involves the septal annulus, which is away from the free wall of the right ventricle, and thus clamping of this site may damage the adjacent tissue (5).

As an emerging TTVI device to correct TR, the preprocedural evaluation parameters, indications, and manipulation process of the K-Clip™ system still need to promote perfection. In addition, the long-term clinical outcome’s durability compared with surgical annuloplasty needs high-quality randomized controlled trials as proof. At present, the K-Clip™ is mainly applicable to elderly and high-risk patients with severe secondary TR. Regarding the implantation of K-Clip™ in patients with recurrent TR who have undergone prior Kay’s annuloplasty, comprehensive and careful evaluation is needed both preprocedurally and intraprocedurally.

## Conclusion

TTVI via the K-Clip™ system is a novel alternative for patients with high surgical risk to correct severe secondary TR. As for symptomatic patients with recurrent TR who have undergone prior Kay’s annuloplasty, the indications for the K-Clip™ procedure are more rigorous, and the evaluation should be more comprehensive and careful.

## Data Availability

The original contributions presented in the study are included in the article/Supplementary Material, further inquiries can be directed to the corresponding author.
